# Comparative Analysis of Anticoagulation Stability in Critically Ill Patients Receiving Argatroban for Suspected or Confirmed Heparin-Induced Thrombocytopenia Compared with Unfractionated Heparin: A Retrospective Cohort Study

**DOI:** 10.3390/hematolrep18030031

**Published:** 2026-04-30

**Authors:** Imran Khan, Elizabeth Lamarche, Bernadett Kovacs, Ariel Hendin, Andy Pan, Caitlin Richler, Christine Landry, Sydney Morin, Kaouther Derouiche, Pierre Thabet

**Affiliations:** Montfort Hospital, Ottawa, ON K1K 0T2, Canadabernadettkovacs@montfort.on.ca (B.K.); arielhendin@montfort.on.ca (A.H.); christine.landry@uottawa.ca (C.L.); kaoutherderouiche@montfort.on.ca (K.D.);

**Keywords:** argatroban, unfractionated heparin, time in therapeutic range, aPTT, heparin resistance, heparin-induced thrombocytopenia, anticoagulation stability, critical care, intensive care unit, retrospective cohort

## Abstract

**Background:** Achieving and maintaining therapeutic anticoagulation with unfractionated heparin in critically ill patients is challenging due to biologic variability, heparin resistance, and limitations of activated partial thromboplastin time (aPTT) monitoring. Argatroban, a direct thrombin inhibitor, provides antithrombin-independent anticoagulation with more predictable pharmacokinetics, but real-world data describing its anticoagulation stability in the intensive care unit (ICU) remain limited. **Objective:** This study aimed to compare anticoagulation stability between continuous intravenous argatroban and unfractionated heparin in critically ill patients using time in therapeutic range (TTR) based on aPTT as the primary performance metric. **Methods:** A retrospective cohort study was conducted in the ICU and step-down unit of Hôpital Montfort (Ottawa, ON, Canada) between January 2016 and December 2024. Adult patients receiving continuous intravenous argatroban or unfractionated heparin for systemic anticoagulation were included. All aPTT values obtained during active infusion were extracted, and TTR was calculated using linear interpolation between consecutive measurements. Continuous variables were summarized as medians with interquartile ranges and compared using the Wilcoxon rank-sum test; categorical TTR strata were compared using Fisher’s exact test. **Results:** Sixty-eight patients met the inclusion criteria, contributing 9 argatroban and 61 heparin infusion courses. Argatroban demonstrated a higher median TTR than heparin (83.3% [IQR 82.0–90.7] vs. 47.5% [32.9–62.4]; *p* < 0.001), with a moderate-to-large effect size (r = 0.51). Median aPTT values were similar between groups, but argatroban showed narrower dispersion and fewer prolonged subtherapeutic periods. A majority of heparin courses (56.5%) spent <50% of time within range, whereas no argatroban courses fell into this category. Conversely, 33.3% of argatroban courses achieved ≥90% TTR compared with none in the heparin group. **Conclusions:** In this real-world ICU cohort where argatroban was used for suspected or confirmed HIT, argatroban was associated with higher TTR than unfractionated heparin. These findings support the use of time-dependent metrics to evaluate anticoagulation quality and warrant prospective studies in more homogeneous populations.

## 1. Introduction

Maintaining therapeutic anticoagulation with unfractionated heparin, as assessed by partial thromboplastin time (PTT), is challenging in clinical practice and particularly in critically ill patients [1]. Heparin therapy is frequently complicated by heparin resistance, in which disproportionately high doses are required to achieve therapeutic anticoagulation [2]. This phenomenon is multifactorial and may reflect antithrombin deficiency, increased heparin clearance, or elevated levels of heparin-binding proteins, contributing to difficulty achieving and sustaining target PTT values [2,3]. As a result, patients may experience prolonged exposure to subtherapeutic anticoagulation with increased thromboembolic risk, or supratherapeutic anticoagulation with increased bleeding risk [3,4].

Heparin-induced thrombocytopenia with thrombosis (HITT) is a serious immune-mediated complication of heparin characterized by thrombocytopenia and paradoxical thrombosis driven by antibodies to platelet factor 4–heparin complexes [5,6]. In patients with suspected or confirmed HIT/HITT, alternative anticoagulants that do not rely on heparin or antithrombin are required. Argatroban, a direct thrombin inhibitor, is commonly used in this setting because it provides antithrombin-independent thrombin inhibition with rapid onset, a short half-life, and predictable pharmacokinetics that facilitate titration and monitoring [7,8]. Argatroban is approved for HIT and for use during percutaneous coronary intervention in patients with HIT, and it is also used in clinical scenarios where heparin response is unreliable and consistent systemic anticoagulation is required [9,10].

Despite these theoretical advantages, data describing the consistency of argatroban anticoagulation control in critically ill patients remain limited [11]. In parallel, interpretation of apparent “heparin resistance” in the ICU is complicated by the distinction between true pharmacologic resistance and pseudo-resistance. True heparin resistance reflects reduced anticoagulant effect despite dose escalation, most commonly due to antithrombin deficiency, increased clearance, or increased heparin-binding proteins [12]. In contrast, pseudo-resistance occurs when therapeutic heparin concentrations are present but the activated partial thromboplastin time (aPTT) remains subtherapeutic due to elevated factor VIII, fibrinogen, or other acute-phase reactants that shorten the aPTT [13]. This laboratory–clinical discordance is common in systemic inflammation and may lead to inappropriate dose escalation when aPTT is used as the sole monitoring assay [13]. Consequently, difficulty achieving therapeutic aPTT targets in critically ill patients may reflect both pharmacologic limitations of heparin and assay-related limitations of aPTT monitoring, underscoring the importance of evaluating anticoagulation quality using time-dependent metrics.

Accordingly, this retrospective cohort study evaluated anticoagulation stability in critically ill patients receiving argatroban compared with unfractionated heparin by quantifying time in therapeutic range (TTR), defined as the proportion of serial aPTT results within the target therapeutic window. Secondary analyses assessed anticoagulation stability by examining aPTT distributions and the proportion of time spent in subtherapeutic and supratherapeutic ranges. By focusing on sustained time within range rather than isolated aPTT values, this study aims to evaluate whether argatroban is associated with more consistent anticoagulation control than heparin in the ICU within a real-world clinical context.

## 2. Methods

### 2.1. Study Objectives

The primary objective of this retrospective study was to compare anticoagulation control in critically ill patients receiving continuous intravenous argatroban with that achieved using unfractionated heparin at our institution, as measured by time in therapeutic range (TTR) based on activated partial thromboplastin time (aPTT). The heparin comparator consisted of a previously derived institutional ICU cohort in which TTR had been calculated using the same monitoring protocol and analytic methodology applied to both anticoagulant groups in the present study.

Secondary objectives included comparison of the proportion of time spent in subtherapeutic and supratherapeutic anticoagulation ranges, characterization of the distribution of aPTT values obtained during active infusion, and identification of clinically documented consequences potentially associated with anticoagulation instability.

### 2.2. Study Design and Population

This was a retrospective cohort study conducted in adult patients (≥18 years) admitted to the intensive care unit (ICU) or step-down (intermediate care) unit at Hôpital Montfort between 1 January 2016 and 31 December 2024.

Eligible patients were identified using institutional pharmacy dispensing records for continuous intravenous argatroban and unfractionated heparin infusions and were verified through detailed electronic medical record review. All aPTT values obtained during active anticoagulant infusion were extracted for both treatment groups, and time in therapeutic range was calculated using the same interpolation-based method.

At our institution, argatroban is reserved for patients with suspected or confirmed heparin-induced thrombocytopenia (HIT); therefore, all patients in the argatroban cohort received argatroban for this indication. The heparin cohort consisted of ICU and step-down patients who received continuous intravenous unfractionated heparin for systemic anticoagulation during the same study period.

Patients were excluded if:

- aPTT measurements were incomplete or unavailable during active anticoagulant infusion.
- A pre-existing coagulation disorder was present that could confound the interpretation of aPTT.
- Systemic anticoagulation was delivered during continuous renal replacement therapy (CRRT) due to the known effect of extracorporeal circuits on coagulation parameters.

### 2.3. Intervention and Comparator

#### 2.3.1. Argatroban Cohort

Patients in the intervention group received continuous intravenous argatroban for systemic anticoagulation. Dosing and infusion adjustments followed the institutional standardized anticoagulation protocol, in which infusion rates are titrated according to serial aPTT measurements obtained at predefined intervals (typically every 4–6 h until therapeutic stability is achieved).

#### 2.3.2. Heparin Comparator Cohort

The comparator consisted of ICU patients who received continuous intravenous unfractionated heparin during the same study period. These patients were identified using the same pharmacy dispensing records and electronic medical record review as the argatroban cohort. All aPTT values obtained while the heparin infusion was actively running were extracted, and time in therapeutic range was calculated using the same linear interpolation method and patient-specific therapeutic targets as for argatroban.

#### 2.3.3. Therapeutic Targets

Both anticoagulants were titrated according to institutional protocols to indication-specific therapeutic aPTT targets (40–60 s or 60–80 s). All TTR calculations were normalized to the prescribed therapeutic range for each infusion.

### 2.4. Outcomes

#### 2.4.1. Primary Outcome

The primary outcome was patient-level time in therapeutic range (TTR), defined as the proportion of total anticoagulation duration during which interpolated aPTT values were within the prescribed therapeutic window.

TTR was calculated using linear interpolation between consecutive aPTT measurements, allowing anticoagulation performance to be assessed as a continuous time-dependent variable rather than as discrete laboratory values.

#### 2.4.2. Secondary Outcomes

Secondary analyses included:

- Proportion of time spent below the therapeutic range.
- Proportion of time spent above the therapeutic range.
- Distribution of aPTT values during active infusion.

When clinically documented, potential consequences of anticoagulation instability—such as thromboembolic events or diagnostic imaging prompted by concern for inadequate anticoagulation—were recorded descriptively. Clinically documented adverse drug reactions, including hypersensitivity reactions, were reviewed descriptively when available in the medical record.

### 2.5. Unit of Analysis and Exposure Definition

For anticoagulation performance analyses, the unit of analysis was the individual infusion course. Each continuous anticoagulant infusion with sufficient aPTT measurements to permit interpolation-based calculation was treated as an independent observation. The start of anticoagulation was defined as the documented time of infusion initiation, and the end as the time of infusion discontinuation. Only aPTT values drawn while the infusion was actively running were included in the analysis.

### 2.6. Data Collection

This study was conducted in the 18-bed ICU and step-down unit of Hôpital Montfort, a 289-bed academic community teaching hospital in Ottawa, Ontario, Canada.

For each eligible patient, the following variables were collected:

- Age.
- Sex.
- Body weight.
- ICU admission diagnosis.
- Indication for systemic anticoagulation.
- Anticoagulant administered.
- Infusion duration.
- All infusion rate adjustments.
- All aptt measurements obtained during active infusion.

All data were extracted through a detailed electronic medical record review and were anonymized at the time of collection.

### 2.7. Statistical Analysis

Continuous variables were summarized as median with interquartile range (IQR) or mean with standard deviation (SD), as appropriate based on distribution. Categorical variables were expressed as counts and percentages.

Normality of continuous variables was assessed using visual inspection of distributions and the Shapiro–Wilk test. Because TTR and aPTT data were not normally distributed, between-group comparisons were performed using the Wilcoxon rank-sum test. Effect size for nonparametric comparisons was reported as the rank-biserial correlation coefficient (r), calculated from the Wilcoxon rank-sum test statistic using standard methods implemented in R statistical software (version 4.3.2, R Foundation for Statistical Computing, Vienna, Austria).

Categorical comparisons of TTR strata were performed using Fisher’s exact test due to small expected cell counts.

The infusion course was defined as the primary unit of analysis for anticoagulation performance. Each infusion course was treated as an independent observation, although a small number of patients contributed more than one course. Given the limited frequency of repeat observations within individual patients, the potential impact of within-patient clustering on overall results was considered minimal, although this was not formally evaluated.

All tests were two-sided, and a *p*-value < 0.05 was considered statistically significant. All statistical analyses were conducted using R statistical software (version 4.3.2, R Foundation for Statistical Computing, Vienna, Austria), with Microsoft Excel (version 2021, Microsoft Corporation, Redmond, WA, USA) used for data management and preprocessing.

### 2.8. Ethics

This study received approval from the Hôpital Montfort Research Ethics Board (REB #: 24-25-10-036) prior to initiation of data extraction and analysis. Given the retrospective design and use of existing health records, a waiver of informed consent was granted.

Data collection was limited to variables required to meet study objectives. All data were anonymized and assigned a study identifier at the time of extraction. No patient-identifiable information was retained. Electronic data were stored on encrypted, password-protected institutional servers accessible only to authorized study personnel, in accordance with institutional privacy policies and applicable Canadian regulations.

## 3. Results

### 3.1. Cohort Identification and Dataset Construction

To provide transparency regarding cohort derivation, we first describe the overall anticoagulant exposure dataset prior to application of study-specific inclusion and exclusion criteria. Between January 2016 and December 2024, we identified 277 anticoagulant prescriptions across 169 unique patients in the overall dataset prior to application of exclusion criteria. Baseline characteristics of this cohort are presented in Table 1. Heparin used in the CRRT setting was consistently prescribed as a single course per patient, whereas intravenous heparin infusions were most commonly limited to one course, with only a small proportion of patients receiving two courses. Argatroban exposure was less frequent and demonstrated greater within-patient variability, ranging from one to three prescriptions. At this stage of dataset construction, 7 patients had received argatroban, 41 had received CRRT heparin, and 121 had received IV heparin. Baseline characteristics of this overall hospital cohort are presented in Table 1.

After application of the pre-specified exclusions, including removal of all CRRT cases and all non-ICU or step-down unit administrations, 73 prescriptions across 68 patients met eligibility criteria for analysis in the ICU setting. This included 7 patients treated with argatroban, contributing 9 infusion courses, and 61 patients treated with IV heparin, contributing 61 infusion courses. The infusion course was defined as the primary unit of analysis for anticoagulation performance. Baseline characteristics of the final ICU analytic cohort, see Table 2, were broadly comparable with respect to age and body weight, although the proportion of male patients was higher in the heparin group. These data are summarized in Table 2. At our institution, argatroban is reserved for suspected or confirmed heparin-induced thrombocytopenia, and all argatroban infusions in this cohort were initiated for this indication.

### 3.2. Aptt Measurements During Active Infusion

**Figure 1:**
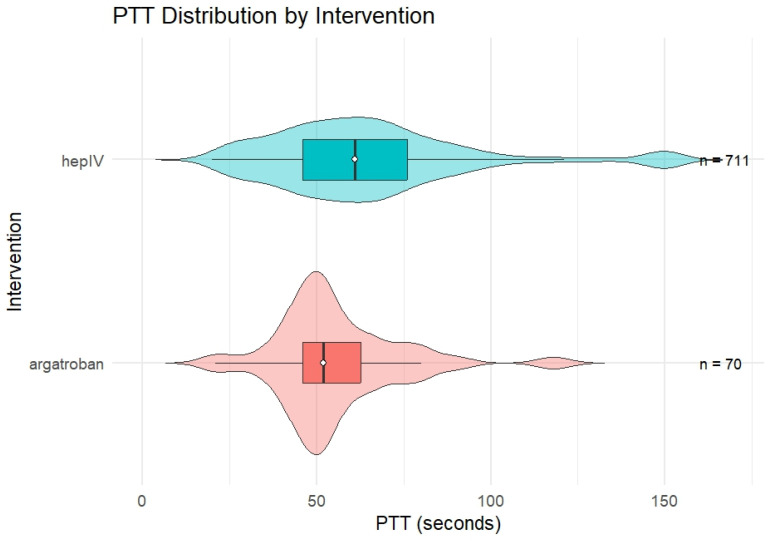
Distribution of aPTT Values During Continuous Argatroban and Unfractionated Heparin Infusion.

Following application of predefined exclusion criteria, including removal of CRRT cases and restriction to ICU and step-down settings, the final analytic cohort was established as follows. During active anticoagulation, the argatroban cohort contributed 70 aPTT measurements, whereas the IV heparin cohort contributed 711 measurements. Argatroban was associated with a lower median aPTT and a narrower interquartile range compared with IV heparin. The median aPTT during argatroban infusion was 52 s (IQR 16.8; range 21–118), compared with 61 s (IQR 30; range 20–150) during IV heparin infusion. Mean ± standard deviation values were 55.4 ± 17.9 s for argatroban and 64.9 ± 28.7 s for IV heparin. Both medians remained within the institutional therapeutic target range. These aPTT summaries are descriptive in nature and reflect repeated measurements obtained during active infusion rather than patient-level aggregates. Detailed aPTT summary statistics are provided in Table 3, and the distribution of aPTT values for each anticoagulant is illustrated in Figure 1.

### 3.3. Time in Therapeutic Range (Ttr): Continuous Analysis

**Figure 2:**
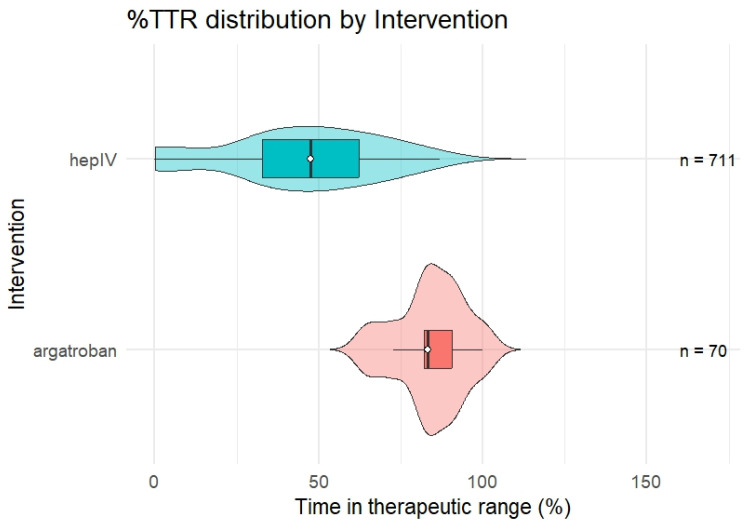
Violin Plot of Patient-Level Time in Therapeutic Range for Argatroban and Unfractionated Heparin.

TTR was calculated for each infusion course, and each course was treated as an independent observation despite some patients contributing more than one course. Argatroban was associated with a higher time in therapeutic range than IV unfractionated heparin. The median TTR for argatroban was 83.3% (IQR 82.0–90.7) compared with 47.5% (IQR 32.9–62.4) for IV heparin, representing an absolute median difference of 35.8%. This difference was statistically significant on Wilcoxon rank-sum testing (*p* < 0.001) and was associated with a moderate-to-large effect size (r = 0.51). In contrast, median aPTT values were similar between groups (52 s [50–67] for argatroban versus 60.5 s [50.2–69.8] for IV heparin; *p* = 0.730), indicating that the primary distinction between agents was the proportion of treatment time spent within the therapeutic range rather than the attainment of isolated therapeutic values. The distribution of TTR as a continuous variable is shown in Figure 2.

### 3.4. Time in Therapeutic Range (Ttr): Category Analysis

**Figure 3:**
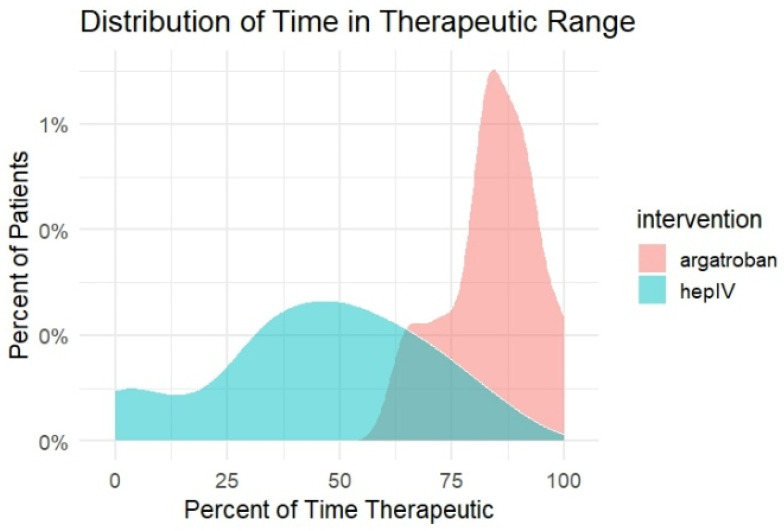
Density Distribution of Time in Therapeutic Range by Anticoagulant.

**Figure 4:**
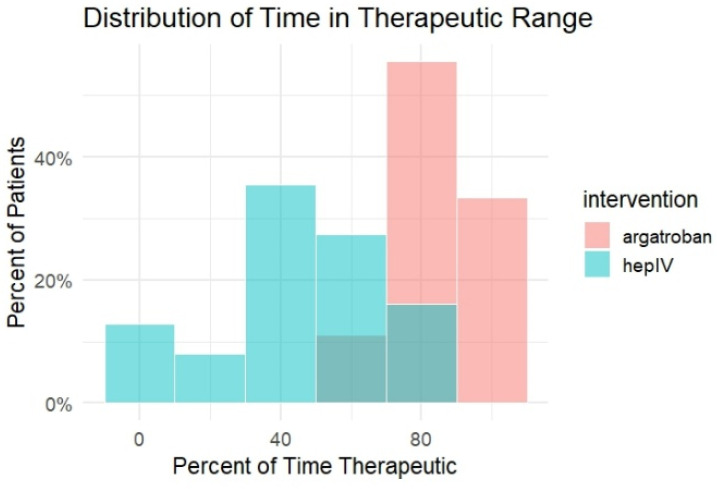
Frequency Distribution of Time in Therapeutic Range by Anticoagulant.

Categorical analysis of TTR demonstrated clear separation between treatment groups. A majority of patients receiving IV heparin (56.5%) spent less than 50% of treatment time within the therapeutic range, whereas no argatroban-treated patients fell into this lowest category. At the upper end of therapeutic control, 33.3% of argatroban-treated patients achieved a TTR of at least 90%, compared with none in the IV heparin group. In the intermediate categories, 44.4% of argatroban patients achieved a TTR of 75–89.9% compared with 9.7% of IV heparin patients, while 22.2% of argatroban patients and 33.9% of IV heparin patients fell within the 50–74.9% range. Absolute differences in the proportion of patients within each TTR category, together with 95% confidence intervals, are presented in Table 4. The density and histogram plots of TTR demonstrate a rightward shift in the argatroban group and greater dispersion with IV heparin, as shown in Figure 3 and Figure 4.

Because of the small argatroban sample size and the presence of sparse cells within the contingency table, Fisher’s exact test was used to compare the overall distribution of TTR categories between groups. The overall comparison was statistically significant (*p* < 0.001), with the greatest separation observed at the extremes of control. Category distributions and absolute differences are presented in Table 4.

### 3.5. Summary of Anticoagulation Performance

After restriction to ICU and step-down patients and exclusion of CRRT, argatroban was associated with tighter dispersion of aPTT values during infusion and a substantially greater proportion of treatment time within the therapeutic range compared with IV unfractionated heparin. Across both continuous and categorical analyses, patients treated with argatroban demonstrated higher levels of therapeutic control and a lower frequency of prolonged subtherapeutic exposure.

## 4. Discussion

This retrospective cohort study evaluated the stability of systemic anticoagulation with argatroban compared with unfractionated heparin in critically ill patients using time in therapeutic range (TTR) as the primary performance metric. After restriction to ICU and step-down patients and exclusion of CRRT, argatroban was associated with a substantially greater proportion of treatment time within the therapeutic aPTT range than IV heparin (median TTR 83.3% vs. 47.5%), with a moderate-to-large effect size. In parallel, aPTT values obtained during argatroban infusion demonstrated a narrower dispersion than those observed with heparin, whereas median aPTT values were similar between groups. Taken together, these findings indicate that the principal difference between the two anticoagulants was not the attainment of isolated therapeutic values but the ability to maintain anticoagulation within range over time. No allergic or hypersensitivity reactions to argatroban were documented in this cohort.

This distinction is clinically and conceptually important. Anticoagulation is inherently a time-dependent therapy, and isolated laboratory values provide limited information about the quality of anticoagulant exposure. In this cohort, both agents were capable of achieving therapeutic aPTT values; however, patients receiving heparin spent prolonged periods outside the target range, most commonly in sub-therapeutic zones, whereas argatroban maintained therapeutic control for a much greater proportion of the infusion duration. These findings support the use of TTR as a more meaningful measure of anticoagulation performance in the ICU, where rapid physiologic fluctuations, inflammation, and evolving organ dysfunction frequently destabilize drug response.

The observed differences are biologically plausible and align with known pharmacologic properties of the two agents. Unfractionated heparin is an indirect anticoagulant whose activity depends on adequate antithrombin availability and is influenced by nonspecific binding to plasma proteins, acute-phase reactants, and endothelial surfaces [1]. In critical illness, reductions in antithrombin activity, increased heparin clearance, and elevations in heparin-binding proteins contribute to an unstable and nonlinear dose–response relationship, often manifesting clinically as difficulty achieving or maintaining therapeutic anticoagulation [14]. Argatroban, by contrast, directly inhibits thrombin at its catalytic site and does so independently of antithrombin. Its anticoagulant effect is therefore less susceptible to the biologic variability associated with systemic inflammation and critical illness, and its linear pharmacokinetics and short half-life permit more precise titration [9,10]. These pharmacologic differences provide a mechanistic explanation for the tighter aPTT distribution and higher TTR observed in the argatroban cohort.

Importantly, argatroban was used exclusively in patients with suspected or confirmed heparin-induced thrombocytopenia at our institution, and the treatment groups were therefore not exchangeable. The results should not be interpreted as demonstrating superiority of argatroban over heparin in an unselected ICU population but rather as showing that, within this real-world cohort, argatroban was associated with more stable laboratory anticoagulation despite being used in a population in whom heparin was no longer considered appropriate. In this context, the magnitude and internal consistency of the TTR difference across both continuous and categorical analyses are notable and suggest that reliable therapeutic control can be achieved in critically ill patients when anticoagulation is delivered through an antithrombin-independent mechanism.

These findings also contribute to the broader discussion regarding optimal monitoring strategies for unfractionated heparin in the ICU. Activated partial thromboplastin time is influenced by multiple factors common in critical illness, including elevations in factor VIII and fibrinogen, and may not accurately reflect heparin pharmacodynamic activity. The wide dispersion of aPTT values and prolonged sub-therapeutic exposure observed in the heparin cohort are consistent with prior observations that aPTT-based monitoring can be unreliable in this population. By evaluating anticoagulation performance as a time-dependent variable rather than as discrete measurements, this study provides a complementary perspective on the limitations of aPTT-guided heparin therapy in critically ill patients.

This study has several important limitations. First, its retrospective and observational design introduces the possibility of selection bias and confounding by indication, as anticoagulant choice was determined by the treating clinician. Because argatroban was reserved for suspected or confirmed HIT, the two groups differed in underlying clinical context and cannot be considered directly comparable. Second, the argatroban sample size was small, reflecting real-world utilization patterns in a community ICU, and this limits statistical precision and generalizability. Third, anticoagulation quality was assessed exclusively using aPTT-based metrics. aPTT is affected by inflammation and other ICU-specific physiologic derangements, and anti-factor Xa activity, antithrombin levels, fibrinogen concentration, and markers of hypercoagulability were not available to more precisely characterize heparin responsiveness or resistance. Fourth, our institution employs two heparin titration protocols with different therapeutic targets, introducing heterogeneity in anticoagulation goals. Additionally, argatroban is typically initiated using a reduced starting dose (e.g., 0.5 mcg/kg/min) in patients with suspected hepatic dysfunction or severe thrombocytopenia, in accordance with local protocols. However, detailed data on hepatic function and protocol-specific dose adjustments were not systematically collected in this retrospective cohort, limiting assessment of their impact on anticoagulation stability. Fifth, we did not evaluate clinical outcomes such as bleeding, thrombosis, transfusion requirements, ICU length of stay, or cost, and therefore, the clinical impact of the observed differences in TTR cannot be directly determined. Antithrombin activity was not routinely measured in this cohort, limiting our ability to distinguish between true heparin resistance due to antithrombin deficiency and pseudo-resistance related to inflammatory changes affecting aPTT. Concomitant use of other antithrombotic or interacting medications was not systematically captured, precluding assessment of potential drug–drug interactions affecting anticoagulation control. In terms of analysis, because some patients contributed more than one infusion course, within-patient correlation could not be fully accounted for; however, repeat exposure was infrequent and unlikely to materially alter the observed magnitude of effect, although this was not formally evaluated. Finally, the pro-coagulant milieu of critical illness may have influenced anticoagulant response independently of drug selection.

Despite these limitations, the coherence of the findings across multiple analytic approaches—including tighter aPTT dispersion, higher median TTR, and a marked reduction in prolonged sub-therapeutic exposure—supports the observation that argatroban was associated with more temporally stable anticoagulation in this cohort. To our knowledge, few studies in critically ill patients have compared argatroban and unfractionated heparin using TTR as the primary measure of anticoagulation performance, and the present analysis suggests that sustained time within range may be a more informative quality metric than isolated therapeutic measurements in this setting.

These results have potential implications for future research and clinical practice. Prospective studies with larger sample sizes, harmonized therapeutic targets, protocolized titration strategies, and multimodal laboratory monitoring—including anti-factor Xa activity and antithrombin levels—are needed to more precisely define heparin responsiveness in the ICU and to determine whether improved TTR translates into reductions in thrombosis, bleeding, or resource utilization. In parallel, evaluation of workflow burden and cost-effectiveness will be essential to identify the clinical scenarios in which the pharmacologic advantages of argatroban justify its use beyond the setting of HIT.

In summary, when anticoagulation performance was assessed as a time-dependent variable, argatroban was associated with a more stable and sustained therapeutic profile than unfractionated heparin in this critically ill cohort. These findings reinforce the importance of evaluating anticoagulation quality over time and support further investigation of targeted anticoagulant strategies in ICU patients in whom reliable therapeutic control is difficult to achieve.

## 5. Conclusions

In this real-world ICU cohort, where argatroban was used for suspected or confirmed HIT, argatroban was associated with a higher time in therapeutic range than unfractionated heparin. These findings support the use of time-dependent metrics to evaluate anticoagulation quality and warrant a prospective study in more homogeneous populations.

## Figures and Tables

**Table 1:** Baseline Characteristics of the Overall Anticoagulant Exposure Cohort.

Characteristic	Argatroban	Heparin IV	Heparin CRRT
Number of Patients	7	121	41
Age (SD) ^1^	69.5 (7.8)	69.3 (16)	59.8 (16.9)
Weight (SD) ^1^	86.4 (11.9)	83.3 (24)	91.4 (25.1)
% Male (*n*)	20% (2)	53.2% (66)	58.5% (24)

^1^ Mean—Values presented as mean ± standard deviation (SD).

**Table 2:** Baseline Characteristics of the ICU Study Cohort.

Characteristic	Argatroban	Heparin IV
Number of Patients	7	61
Age (SD) ^1^	69.5 (7.8)	65.2 (15.9)
Weight (SD) ^1^	86.4 (11.9)	85 (20.6)
% Male (*n*)	20% (2)	63.5% (40)

^1^ Mean—Values presented as mean ± standard deviation (SD).

**Table 3:** Distribution of aPTT Values During Continuous Anticoagulant Infusion.

Intervention	# Patients	# Samples	Median	IQR	Min	Max	Mean	SD
Argatroban	7	70	52	16.8	21	118	55.4	17.9
Heparin IV	62	711	61	30	20	150	64.9	28.7

**Table 4:** Distribution of Time in Therapeutic Range (TTR) Categories by Anticoagulant.

% Time in Therapeutic Range	Heparin IV (*n*, %)	Argatroban (*n*, %)	Difference [95% CI]	Fisher Exact Test (*p*-Value)
0–49.9%	35 (56.5%)	0 (0%)	−56.5% [−75.2%, −37.7%] *	<0.001
50–74.9%	21 (33.9%)	2 (22.2%)	−11.6% [−47.6%, 24.3%] *	
75–89.9%	6 (9.7%)	4 (44.4%)	34.8% [−4.9%, 74.4%] *	
≥90%	0 (0%)	3 (33.3%)	33.3% [−3.8%, 70.5%] *	

* Asterisk indicates CI may be unreliable due to small counts (*n* < 5). Comparison of TTR category distributions between groups performed using Fisher’s exact test.

## Data Availability

The original contributions presented in this study are included in the article. Further inquiries can be directed to the corresponding author.
